# Antibodies from Sierra Leonean and Nigerian Lassa fever survivors cross-react with recombinant proteins representing Lassa viruses of divergent lineages

**DOI:** 10.1038/s41598-020-72539-w

**Published:** 2020-09-29

**Authors:** Megan L. Heinrich, Matthew L. Boisen, Diana K. S. Nelson, Duane J. Bush, Robert W. Cross, Anatoliy P. Koval, Andrew R. Hoffmann, Brandon J. Beddingfield, Kathryn M. Hastie, Megan M. Rowland, Irina Aimukanova, Sophia Koval, Raju Lathigra, Viktoriya Borisevich, Mambu Momoh, John Demby Sandi, Augustine Goba, lkponmwosa Odia, Francis Baimba, John O. Aiyepada, Benevolence Ebo, Philomena Eromon, Chinedu Ugwu, Onikepe Folarin, Testimony Olumade, MacDonald N. Onyechi, Johnson Etafo, Rashidat Adeyemi, Elijah E. Ella, Maryam Aminu, Simji S. Gomerep, Matthew Afam Eke, Olusola Ogunsanya, George O. Akpede, Danny O. Asogun, Sylvanus A. Okogbenin, Peter O. Okokhere, Johan Holst, Jeffrey G. Shaffer, John S. Schieffelin, Thomas W. Geisbert, Erica Ollmann Saphire, Christian T. Happi, Donald S. Grant, Robert F. Garry, Luis M. Branco

**Affiliations:** 1 Zalgen Labs, LCC, Germantown, MD USA; 2 Department of Microbiology and Immunology, University of Texas Medical Branch, Galveston, TX USA; 3 Galveston National Laboratory, Galveston, TX USA; 4 Department of Microbiology and Immunology, School of Medicine, Tulane University, 1430 Tulane Avenue, New Orleans, LA JBJ56870118 USA; 5 La Jolla Institute for Immunology, La Jolla, CA 92037 USA; 6 Department of Pathology, University of Texas Medical Branch, Galveston, TX USA; 7 Eastern Polytechnic Institute, Kenema, Sierra Leone; 8 Viral Hemorrhagic Fever Program, Kenema Government Hospital, Kenema, Sierra Leone; 9 Ministry of Health and Sanitation, Freetown, Sierra Leone; 10 Institute of Lassa Fever Research and Control, Irrua Specialist Teaching Hospital, Irrua, Edo State Nigeria; 11 The African Center of Excellence for Genomics of Infectious Diseases, Redeemer’s University, Ede, Osun State Nigeria; 12 Department of Biological Sciences, College of Natural Sciences, Redeemer’s University, Ede, Osun State Nigeria; 13 Federal Medical Center Owo, Owo, Nigeria; 14 Ahmadu Bello University, Zaria, Nigeria; 15 University Teaching Hospital, Jos, Nigeria; 16 Federal Medical Center, Abakaliki, Nigeria; 17 University of Ibadan, Ibadan, Nigeria; 18 Department of Paediatrics, Irrua Specialist Teaching Hospital, Irrua, Nigeria; 19 Department ofPaediatrics, College of Medicine, Ambrose Alli University, Ekpoma, Nigeria; 20 Department of Community Medicine, Irrua Specialist Teaching Hospital, Irrua, Nigeria; 21 Department of Obstetrics and Gynaecology, Irrua Specialist Teaching Hospital, Irrua, Nigeria; 22 The Department of Medicine, Irrua Specialist Teaching Hospital, Irrua, Edo State Nigeria; 23 The Department of Medicine, Faculty of Clinical Sciences, Ambrose Alli University, Ekpoma, Edo State Nigeria; 24 CEPI (Coalition for Epidemic Preparedness Innovations), Oslo, Norway; 25 Department of Biostatistics and Bioinformatics, Tulane School of Public Health and Tropical Medicine, New Orleans, LA USA; 26 Sections of Infectious Disease, Departments of Pediatrics and Internal Medicine, School of Medicine, Tulane University, New Orleans, LA USA; 27 Department of Molecular Biology and Genomics, Center of Excellence for Genomics of Infectious Diseases (ACEGID), Redeemer’s University, Ede, Osun State Nigeria

**Keywords:** Immunology, Microbiology

## Abstract

Lassa virus (LASV) is the causative agent of Lassa fever, an often-fatal hemorrhagic disease that is endemic in West Africa. Seven genetically distinct LASV lineages have been identified. As part of CEPI’s (Coalition for Epidemic Preparedness Innovations) Lassa vaccine development program, we assessed the potential of the human immune system to mount cross-reactive and cross-protective humoral immune responses to antigens from the most prevalent LASV lineages, which are lineages II and III in Nigeria and lineage IV in Sierra Leone. IgG and IgM present in the blood of Lassa fever survivors from Nigeria or Sierra Leone exhibited substantial cross-reactivity for binding to LASV nucleoprotein and two engineered (linked and prefusion) versions of the glycoproteins (GP) of lineages II–IV. There was less cross-reactivity for the Zinc protein. Serum or plasma from Nigerian Lassa fever survivors neutralized LASV pseudoviruses expressing lineage II GP better than they neutralized lineage III or IV GP expressing pseudoviruses. Sierra Leonean survivors did not exhibit a lineage bias. Neutralization titres determined using LASV pseudovirus assays showed significant correlation with titres determined by plaque reduction with infectious LASV. These studies provide guidance for comparison of humoral immunity to LASV of distinct lineages following natural infection or immunization.

## Introduction

Lassa virus (LASV) is the causative agent of Lassa fever, an often fatal viral hemorrhagic fever (VHF). Cases are reported year round in Nigeria, Sierra Leone, Liberia, Guinea and other West African countries, with peak incidence in the dry season. Case-fatality rates (CFRs) among hospitalized Lassa fever patients vary from approximately 25% in Nigeria^1,2^ to greater than 60% in Sierra Leone^3^. Subclinical infections appear to be common^4,5^. A variety of factors, including differential virulence of LASV strains and variations in human genetic susceptibility, immune responses or patient care, may account for the range of CFRs. The main reservoir of LASV is *Mastomys natalensis*, the natal multimammate rat (or mouse), an abundant peridomestic rodent^6–8^. Additional rodent reservoirs or intermediate hosts have been discovered^8,9^. While human-to-human transmission can occur, especially in hospital settings, most infections occur by exposure to rodent excreta or during preparation of rodents for food. Accurate estimates for the number of Lassa fever cases and deaths are not possible because of the limited availability of epidemiological data. Supportive care including management of fluid and electrolyte balance can improve survival^10,11^. The only available treatment is the off-label use of the nucleoside drug ribavirin^12^. There is currently no approved Lassa fever vaccine. Lassa fever has been recognized by the World Health Organization (WHO) as an important threat to global health that is in urgent need of countermeasure development^13^. The Coalition for Epidemic Preparedness Innovations (CEPI) has prioritized the accelerated development of a Lassa fever vaccine^14,15^. CEPI has also initiated an epidemiological study in five West African countries as part of their strategy to facilitate vaccine development efforts^16^.

LASV is a single-stranded RNA virus in the family *Arenaviridae (*Order: *Bunyavirales*)^17^. Its genome consists of two ambisense segments encoding four proteins: Z (zinc, matrix), L (polymerase), NP (nucleoprotein), and GPC (glycoprotein complex)^18^. GPC is post-translationally cleaved into glycoprotein 1 (GP1), glycoprotein 2 (GP2), and a stable signal peptide (SSP) that trimerize on the virion surface^19^. There are currently seven proposed genetically distinct LASV lineages (lineages I–VII) distributed throughout West Africa^20^. Phylogenetic analyses of the highly divergent LASV genomes suggest that the virus has been circulating in Nigeria for over a thousand years, followed by a more recent spread across West Africa^21^. LASV lineages and sublineages (clades) cluster geographically suggesting that once established in a region the virus remains stably separated in the rodent reservoirs^22–24^. The initial isolate of LASV representing lineage I has been detected in northern Nigeria^25^, but has rarely been observed in recent samplings^20,23,26^. lineages II and III are the most common lineages in Nigeria and are found in the southern and central regions, respectively^27,28^. Recently, lineages and phylogenetic clusters in Nigeria have been geographically mapped at a higher resolution^20^. Within lineage II five sublineages were distinguishable. Seven sublineages were distinguishable in lineage III. Lineage IV LASV, including the prototypical Josiah strain commonly used in laboratory studies, is present in Sierra Leone, Liberia and Guinea^21,29^. LASV lineage V is found in Mali and Ivory Coast^30,31^. LASV isolates from the *Hylomyscus pamfi* rodent trapped in Nigeria^8^ and from a nosocomial outbreak in Togo^32^ have been proposed to represent new lineage VI and lineage VII, respectively.

Development of Lassa fever countermeasures is potentially challenged by the high genetic diversity of LASV^14,33^. The genetic variability of LASV could produce differences in the antigenicity of LASV proteins^34–36^. It is unclear whether a vaccine produced with antigens of LASV from a particular lineage will induce a human immune response that is protective against LASV of other lineages. Most Lassa vaccines in development have employed antigens of the Josiah strain (LIV) of LASV^37,38^. As a component of CEPI’s efforts to facilitate and accelerate Lassa vaccine development, serum or plasma from Lassa fever survivors living in different geographical areas and thus representing different lineages has been collected through a contractual arrangement between CEPI and the Viral Hemorrhagic Fever Consortium (VHFC). Vaccine developers are being supplied with panels consisting of samples with high, medium and low titres from survivors of infection with different LASV lineages. These samples in addition to Lassa negative control samples are being used to establish and fine-tune immunoassays for vaccine development.

A suite of Lassa fever diagnostic immunoassays has been developed based on LASV recombinant proteins, including antigen- IgG- and IgM-capture ELISA and rapid diagnostic tests (RDTs)^39–43^. The antibody-capture ELISA has demonstrated the capability to detect IgM seropositivity and subsequent class-switching to IgG seropositivity in Lassa fever cases^41^ and has proven useful in surveillance and vaccine studies^30,44–47^. These prior assays were configured with recombinant antigens based on the lineage IV LASV Josiah strain^39^. To quantify seroreactivity to genetically diverse LASV strains recombinant NP, GP and Z proteins representing additional prominent West African lineages, Nigerian lineages II and III, have been cloned and molecularly characterized. Representative strains from lineage II (LASV237-Nigeria-2010H) and lineage III (LASV-Nig08-A18-Nigeria-2008H) were chosen for molecular cloning and expression based on their approximate median divergence from the corresponding lineage root^27^. Here, we assess the ability of antibodies from Nigerian Lassa fever survivors exposed to LASV lineages II and III and Sierra Leonean Lassa fever survivors exposed to LASV lineage IV to cross-react with recombinant NP, GP and Z proteins representing LASV of these divergent lineages and to cross-neutralize pseudoviruses expressing LASV GPC of these divergent lineages.

## Results

### IgG and IgM capture ELISA based on recombinant antigens representing LASV of divergent lineages

**Figure 1:**
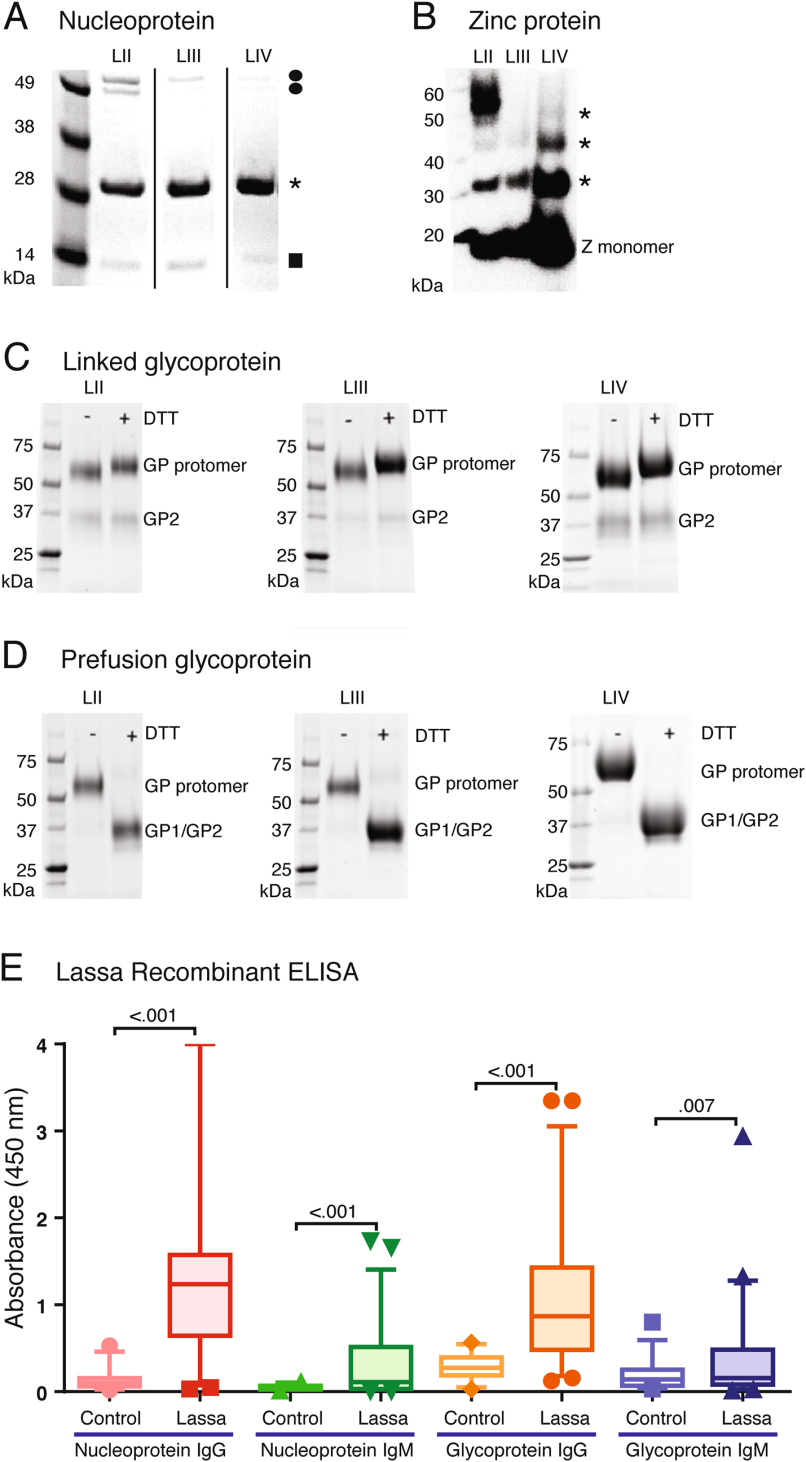
Recombinant LASV proteins representing lineages II–IV and their use in ELISA. Panel (**A**) Nucleoproteins (NP) from LASV representing lineages II–IV were expressed in *E. coli*, purified and resolved by sodium dodecyl-sulfate polyacrylamide gel electrophoresis (SDS-PAGE). Vertical lines indicate removal of lanes from a single gel. Circles: minor contaminants. Asterisk: monomeric NP. Square: NP breakdown product. Panel (**B**): LASV Zinc protein was expressed in *E. coli*, purified and resolved by SDS-PAGE and analyzed by western blotting. Monomeric and multimeric forms of the protein (asterisks) are detected. Panel (**C**) LASV linked glycoprotein was expressed in Drosophila S2 cells, and purified and resolved by SDS-PAGE in the presence and absence of dithiothreitol (DTT). Because most of the GP1 and 2 subunits are covalently linked, reduction by DTT does not affect the mobility of the GP protomer. Panel (**D**): LASV prefusion glycoprotein (Pf-GP) was expressed in Drosophila S2 cells, purified and resolved by SDS-PAGE in the presence and absence of dithiothreitol (DTT). Reduction by DTT converts GP protomer to GP1 and 2 subunits that have similar molecular weights. Panels (**C**,**D**) were prepared from three separate gels. Uncropped polyacrylamide gels and the western blot used to prepare panels (**A**–**D**) are shown in Fig. S1. Panel (**E**): ReLASV Pan-Lassa NP ELISA configured with a mixture of recombinant NP from LASV representing lineages II–IV (LII–IV) and Pan-Lassa Pf-GP ELISA configured with a mixture of recombinant Pf-GP from LASV representing lineages II–IV were used to compare reactivity of IgG and IgM from control samples from heathy United States blood donors (n = 28) versus and Sierra Leonean Lassa fever survivors (n = 58). Data was analyzed using Prism (version 6.07, GraphPad Software, Inc., San Diego, CA) and the figure was compiled using Adobe Illustrator (version 15.1.0, San Jose, CA). Box and whisker plots representing means and standard deviations of samples are indicated. P values for pairwise comparisons are indicated.

Recombinant NP N- and C-terminal domains and Z proteins from lineages II–IV were expressed in codon-optimized *E. coli* strains and purified. The proteins were then visualized on SDS-PAGE and immunoblotting for identity and purity for use as coating antigens for ELISA (Fig. 1A,B, Fig. S1A,B). The NP N- and C-terminal domains from the three lineages are homogenous monomers (Fig. 1A). Minor levels of contaminants and breakdown products are present in the purified NP preparations. In contrast, Z proteins exhibit multimerization; lineage II Z protein forms higher order multimers than Z proteins of lineages III and IV (Fig. 1B).

Two forms of the LASV glycoprotein are utilized in the current studies. Linked LASV GP tethers the GP1 and GP2 subunits together via a flexible linker and is designed to present both pre- and post-fusion epitopes (Fig. 1C, Fig. S1C–F, Fig. S2A). Linked GP has been successfully used for the identification and characterization of anti-LASV GP antibodies from human survivors of Lassa virus infection^48^. Prefusion, stabilized GP is a disulphide linked LASV GP used in the determination of the only pre-fusion structures available for any arenavirus glycoprotein trimer^49,50^. Pre-fusion GP (Pf-GP) contains only the prefusion form (Fig. 1D, Fig. S1C–F, Fig. S2B) and is best suited for detection of serum/plasma IgG and IgM against the complex, neutralizing, domain-spanning epitopes that are a desired product of vaccination efforts. Linked GP and Pf-GP are expressed in Drosophila S2 cells and purified via streptactin-affinity chromatography. Following strepII tag removal with enterokinase, the GPs are further purified by size-exclusion chromatography (SEC). Both linked GP and Pf-GP show appropriate reactions with human monoclonal antibodies (huMAbs) that react with divergent epitopes (Fig. S2C,D)^48^.

A mixture of NP or Pf-GP GP from LASV lineages II, III, and IV immobilized in the ELISA microwell plates was used for the direct absorption of LASV-specific IgM or IgG antibodies from serum or plasma of Lassa fever survivors. Pan-Lassa NP or Pf-GP IgG- and IgM-capture ELISAs exhibited significant differences in reactivity by serum from Sierra Leone Lassa fever survivors compared to serum from United States controls (healthy blood donors) (Fig. 1E). IgG seroreactivity to NP and Pf-GP was higher than IgM seroreactivity in the Sierra Leonean sample cohort. Additional studies on validation of the Pan-Lassa antibody and antigen capture assays is presented elsewhere^51^.

### Nigerian and Sierra Leone Lassa fever survivors reactivity to LASV recombinant antigens

During Jan. 14th–29th, 2019 laboratory staff of Redeemer’s University (RUN), Ede State VHF Laboratory in Nigeria and the Kenema Government Hospital (KGH) VHF Laboratory in Sierra Leone, with training and assistance from Zalgen Labs personnel, conducted studies on seroreactivity to LASV recombinant proteins. Nigerian laboratory personnel from Irrua Specialist Teaching Hospital (ISTH) in Edo State, Federal Medical Center (FMC) in Owo, Ondo State, FMC Abakaliki in Ebonyi State and Ibadan U. were trained to use the ReLASV immunoassays. Lassa fever survivor, contacts, and suspected Lassa fever samples were obtained from ISTH, FMC Owo, and FMC Abakaliki for the study. At KGH post-acute, convalescent and Lassa fever survivor samples were selected from a biorepository of samples obtained from other on-going studies.

**Figure 2:**
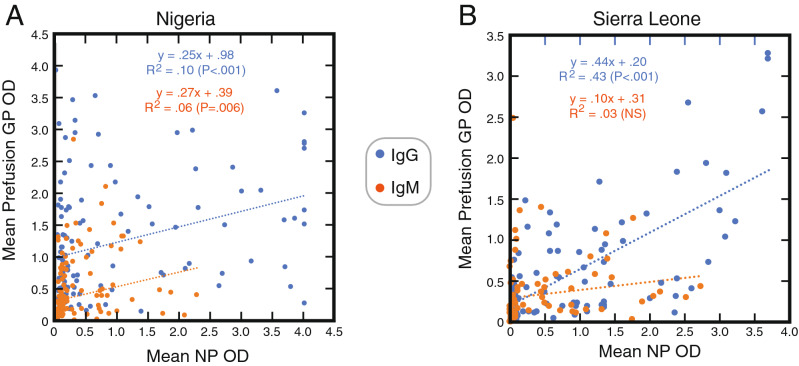
Screening of plasma and serum from Lassa fever survivors for IgG and IgM reactivity to nucleoproteins and glycoprotein from LASV representing lineages II, III and IV. Binding of IgG or IgM in serum or plasma samples from Nigerian (Panel **A**, n = 140) or Sierra Leonean (Panel **B**, n = 80) Lassa fever survivors (1:100 dilution) to nucleoprotein (NP) or Prefusion glycoprotein (Pf-GP) was quantified using the Pan-Lassa NP or Pan-Lassa Prefusion-GP ELISA. Data was analyzed using Microsoft Excel (version 16.39, Microsoft, Redmond, WA) and JMP software (version 13.0.0, SAS Institute, Inc., Cary, NC). The figure was compiled using Adobe Illustrator (version 15.1.0, San Jose, CA). Blue dotted lines are linear regression plots of IgG seroreactivity of a mixture of NP representing lineages II–IV versus a mixture of Pf-GP representing lineages II–IV. Orange dotted lines are linear regression plots comparing IgM seroreactivity for the same mixtures.

Samples were screened using the ReLASV Pan-Lassa NP IgG/IgM ELISA Kit and the prototype ReLASV Pan-Lassa Prefusion GP IgG/IgM ELISA Kit. In Nigeria 140 plasma or serum samples were screened at a 1:100 sample dilution using mixed NP or mixed Prefusion GP ELISAs for IgG/IgM reactivity (Fig. 2). In Sierra Leone 80 plasma and serum samples were selected and screened at a 1:100 sample dilution for IgG and/or IgM reactivity. A range of reactivities to LASV NP and GP was observed in the prescreening of samples from both Sierra Leonean and Nigerian subjects. With the exception of IgM in Sierra Leoneans the correlations between NP and GP reactivity were significant. While many samples had both strong IgG and/or IgM reactivity to both NP and GP other samples had low reactivity to one protein or the other. Linear correlations between NP and GP reactivity in both Sierra Leonean and Nigerian subjects for IgG and IgM had slopes that diverged from 1 (0.10x to 0.44x) indicating lower overall reactivity to GP.

### Binding to antigens encoded by LASV of lineages II, III, and IV

**Figure 3:**
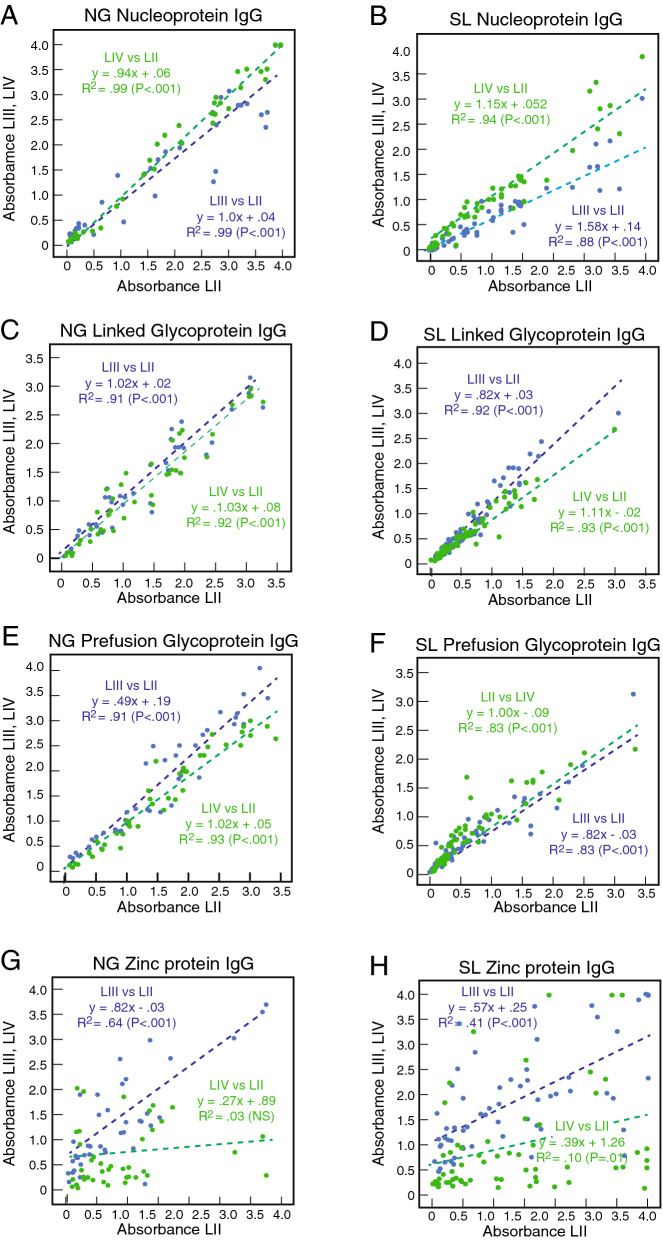
Cross-reactivity of IgG in plasma and serum from Lassa fever survivors for recombinant proteins from LASV of lineages II–IV. Binding of IgG in serum or plasma samples (1:100 dilution) from Nigerian (NG, Panels **A**,**C**,**E**,**G**; n = 40) or Sierra Leonean (SL, Panels **B**,**D**,**F**,**H**; n = 61) Lassa fever survivors quantified using ELISA coated individually with NP, linked GP, Pf-GP or Z from LASV representing lineages II–IV (LII, LIII, LIV) as indicated. Data was analyzed using Microsoft Excel (version 16.39, Microsoft, Redmond, WA) and JMP software (version 13.0.0, SAS Institute, Inc., Cary, NC). The figure was compiled using Adobe Illustrator (version 15.1.0, San Jose, CA). Green dotted lines are linear regression plots of seroreactivity of LASV lineage II antigens versus lineage III antigens. Blue dotted lines are linear regression plots of seroreactivity of lineage II LASV antigens versus lineage IV antigens.

We next assessed a subset of plasma or serum samples from Nigerian (n = 40) and Sierra Leonean (n = 61) with a range of immune responses (from high to low or negative) using ELISAs coated with single antigens, NP, linked GP, Pf-GP, or Z representing LASV of lineages II–IV. Results of the binding studies using a 1:100 sample dilution are presented as scatterplots of the absorbance with linear correlations (Fig. 3). Overall substantial cross-reactivity for IgG binding exists between both NP and linked GP or Pf-GP of LASV of lineages II–IV (Fig. 3A–F). The slopes for all cross-reactivity comparisons were near 1 (range from 0.80x to 1.15x) with the exception of lineage II Pf-GP versus lineage IIwI Pf-GP (slope = 0.49x, Fig. 3E). Binding of plasma or serum IgG to NP and both forms of GP across lineages gave significant R^2^ values (> 0.80, P < 0.001) indicating that there were few samples that bound strongly to protein of one lineage, but weakly to the protein from another lineage.

**Figure 4:**
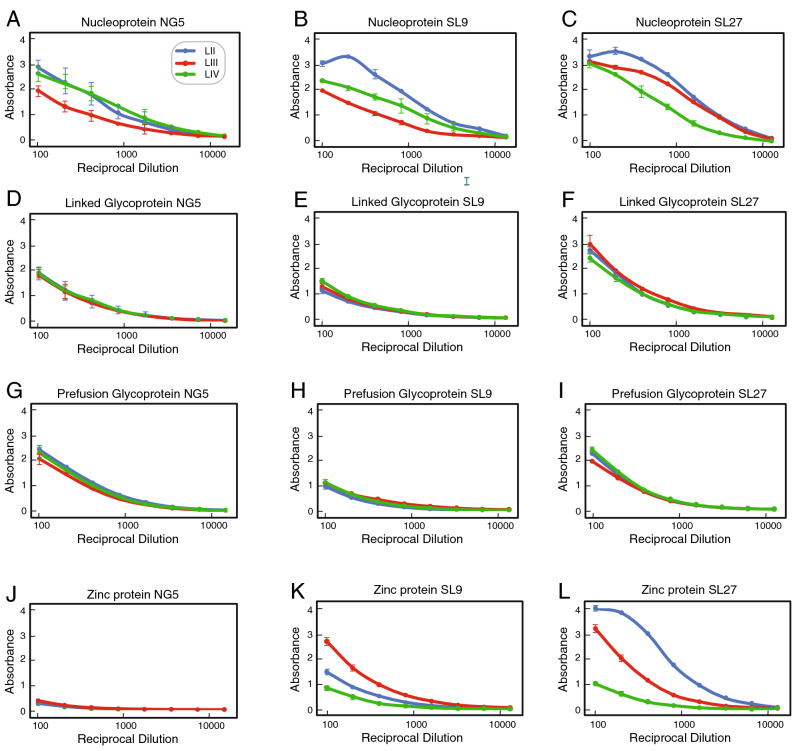
Plasma or serum endpoint dilutions demonstrating cross-reactivity of recombinant proteins from LASV of lineages II–IV. Examples of endpoint dilutions of serum or plasma samples from Nigerian or Sierra Leonean Lassa fever survivors demonstrating reactivity antigens representing LASV lineages II–IV (LII, LII, LIV). Panels **A**,**D**,**G**,**J**: Nigerian survivor 5 (NG5). Panels **B**,**E**,**H**,**K**: Sierra Leonean survivor 9 (SL9). Panels **C**,**F**,**I**,**L**: Sierra Leonean survivor 9 (SL9). ELISA were coated individually with NP, linked GP, Pf-GP or Z from LASV lineages II–IV as indicated. Data was analyzed using Microsoft Excel (version 16.39, Microsoft, Redmond, WA) and JMP software (version 13.0.0, SAS Institute, Inc., Cary, NC). The figure was compiled using Adobe Illustrator (version 15.1.0, San Jose, CA). Blue symbols: lineage II. Red symbols: lineage III. Green symbols: lineage IV. Error bars represent standard deviations, which in most cases were smaller than the symbols.

**Figure 5:**
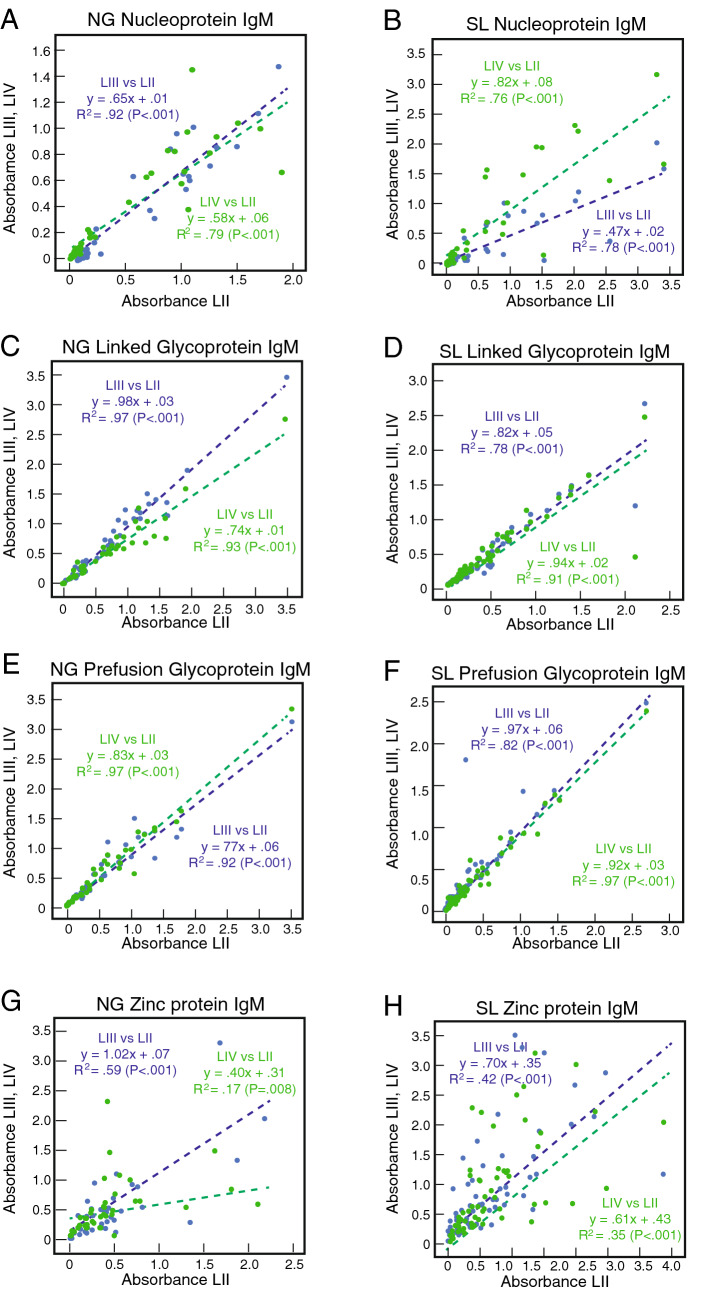
Cross-reactivity of IgM in plasma and serum of Lassa fever survivors for recombinant proteins from LASV of lineages II–IV. Cross-reactivity study for IgM binding conducted as in Fig. 3.

In contrast to the results with NP and GP we found that there was less IgG cross-reactivity between Z proteins of lineages II–IV (Fig. 3G,H). Slopes of 0.82x and 0.57x were observed for comparison of binding of lineage II Z versus lineage III Z for Nigerian and Sierra Leonean samples, respectively. However, for the comparison of lineage IV Z versus lineage II Z the slopes were 0.27x and 0.39x indicating a bias for binding of the lineage II protein. Similar results were obtained with the Z reactivity of Sierra Leonean plasma samples. The differences in the Z protein multimerization may contribute to these differences. Lineage II Z tended to form higher order multimers that may display epitopes more effectively (Fig. 1B). Z is a small protein with a limited number of epitopes that appear to be poorly cross-reactive. Similar correlations for IgG cross-reactivity to NP, linked GP, Pf-GP, or Z representing LASV of lineages II–IV were obtained using endpoint titres rather than quantifying absorbance at a 1:100 dilution (Fig. S3). Typical examples of the reactivities in serial dilution is shown for two Nigerian subjects and one Sierra Leonean subject (Fig. 4). Endpoint titres to LASV NP were typically higher than those to either linked GP, Pf-GP or Z. IgM cross-reactivity among lineages II-IV followed a similar pattern to that observed with IgG (Fig. 5). Class switching from IgM to IgG reactivity is delayed in Lassa fever survivors, which is reflected in the high levels of IgM detected^41^.

### Cross-neutralization studies with LASV pseudoviruses expressing GPC of LASV lineages II–IV

**Figure 6:**
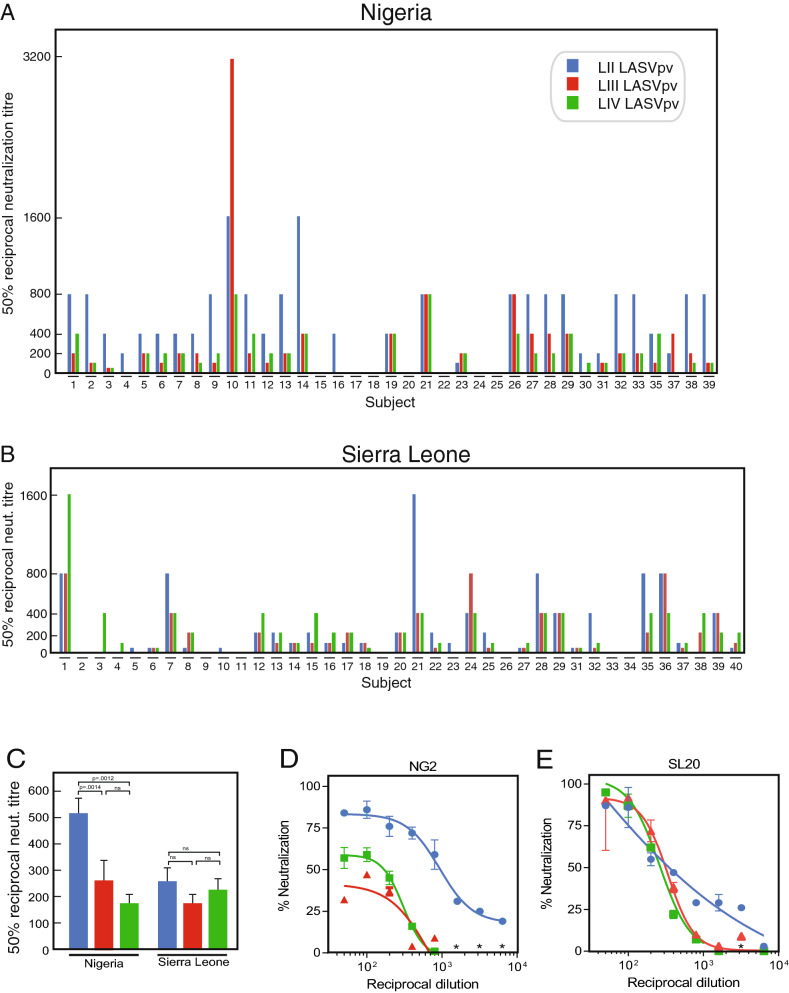
Neutralization of pseudoviruses expressing Lassa virus glycoprotein complex representing lineages II, III and IV by plasma or serum samples of Lassa fever survivors. 50% reciprocal neutralization titres of serum or plasma from Nigerian and Sierra Leonean Lassa fever survivors were determined using LASV pseudoviruses (LASVpv) expressing LASV glycoprotein complexes representing lineages II–IV (LII, LII, LIV). Panel (**A**): Lassa fever survivors from Nigeria (n = 37). Panel (**B**): Lassa fever survivors from Sierra Leone (n = 39). Note that not all samples from the cohorts had sufficient volume to perform the three pv assays. Panel (**C**): Mean of the 50% reciprocal neutralization titres represented in panels (**A**,**B**). Panel (**D**): neutralization curve for Nigerian subject 2 (NG2). Panel (**E**): neutralization curve for Sierra Leonean subject 20 (SL20). Blue bars or symbols are pp representing lineage II. Red bars or symbols are pv representing lineage III. Green bars or symbols are pv representing lineage IV. Data was analyzed using Microsoft Excel (version 16.39, Microsoft, Redmond, WA), JMP software (version 13.0.0, SAS Institute, Inc., Cary, NC) and Prism (version 6.07, GraphPad Software, Inc., San Diego, CA). The figure was compiled using Adobe Illustrator (version 15.1.0, San Jose, CA).Error bars represent standard error of the mean, which is smaller than the symbols in some cases. Asterisks in panels (**D**,**E**) represent enhancement at the indicated dilutions.

Pseudoviruses expressing the GPC of LASV from lineages II, III or IV were employed to assess the ability of antibodies from Lassa fever survivors to cross-neutralize different lineages of LASV (Fig. 6). The average reciprocal 50% neutralization titres were higher in Nigerians than in Sierra Leoneans (Fig. 6A–C). Neutralization by Nigerian samples was biased towards LASVpv lineage II. Reciprocal 50% neutralization titres against LASVpv lineage II were higher than titres against LASVpv expressing lineage III or IV in 23/30 (77%) Nigerian subjects (Fig. 6A). This does not appear to be due to LASVpv lineage II being easier to neutralize by human antibodies. 6/32 Sierra Leonean subjects (13%, p < 0.0001 Fisher’s exact test) had reciprocal 50% neutralization titres against LASVpv lineage II that were higher than the titres for LASVpv lineage III or IV (Fig. 6B). Samples from Sierra Leoneans did not exhibit a strong bias to neutralization of LASVpv lineage IV, their cognate lineage. Only 7/32 samples from Sierra Leonean subjects had reciprocal 50% neutralization titres against LASVpv lineage IV that were higher than those against LASVpv lineage II or III. Eleven of 32 Sierra Leonean samples neutralized LASVpv lineage II or III as well or better than LASVpv LIV. In contrast, only 2/30 samples from Nigerian subjects neutralized LASVpv lineage III or IV as well as they neutralized LASVpv lineage II (p < 0.01).

Neutralization curves for LASV expressing GPC of LII–IV had similar slopes even when there were differences in the reciprocal 50% neutralization titres for the distinct lineages (Fig. 6C–E). Some samples failed to neutralize 100% of any lineage at any dilution tested (Fig. 6C). The failure to neutralize 100% of viruses even at high antibody levels has been noted in prior monoclonal antibody studies^50^. Certain dilutions of serum or plasma resulted in increased infectivity of the pseudovirus (asterisks in Fig. 6D,E). The possible presence of enhancing antibodies in serum from Lassa fever survivors has previously been reported^52^.

**Figure 7:**
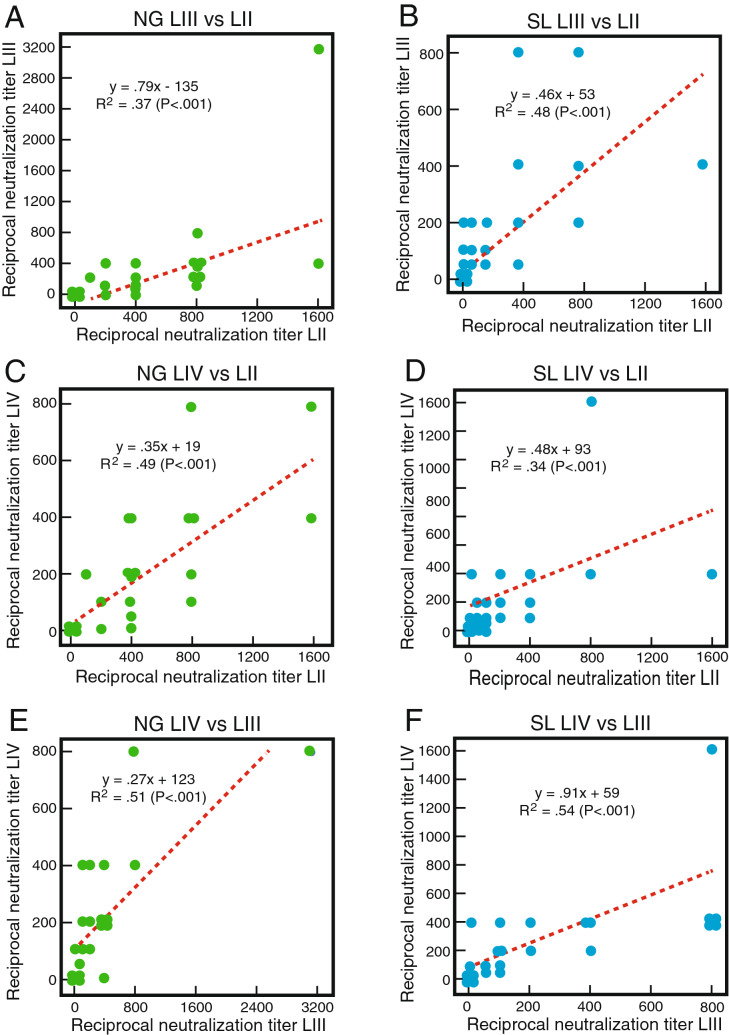
Cross-neutralization by plasma or serum samples of Lassa fever survivors. Linear correlations (dotted red lines) for the neutralization of LASV pseudoviruses expressing LASV glycoprotein complexes representing lineages II, III and IV (LII, LIII and LIV) are indicated. Panels (**A**,**C**,**E**): Nigerian (NG) Lassa fever survivors (n = 37). Panels (**B**,**D**,**F**): Sierra Leonian (SL) Lassa fever survivors (N = 39). Data was analyzed using Microsoft Excel (version 16.39, Microsoft, Redmond, WA) and JMP software (version 13.0.0, SAS Institute, Inc., Cary, NC). The figure was compiled using Adobe Illustrator (version 15.1.0, San Jose, CA). Note that multiple samples had the same 50% neutralization titres for a pair of lineages producing overlapping data points.

Cross-neutralization in neutralizing responses by Nigerian and Sierra Leonean plasma or serum samples was examined using linear correlations (Fig. 7). This analysis confirmed the bias for neutralization of LASVpv lineage II by Nigerian samples (Fig. 7A,C,E). In contrast, bias for neutralization of LASVpv of lineage IV was not demonstrated by Sierra Leonean samples (Fig. 7B,D,F).

### Comparison of recombinant binding assays to pseudovirus neutralization assays and plaque reduction assays using BSL-4 LASV

**Figure 8:**
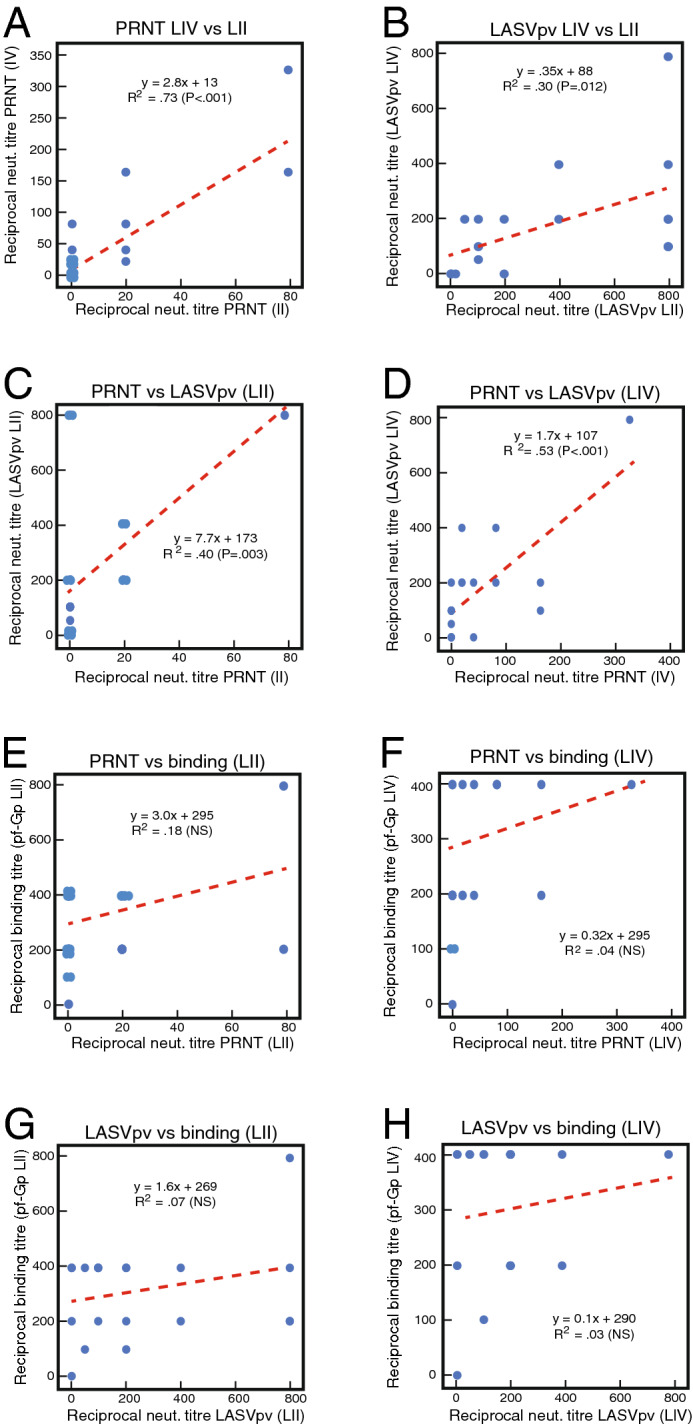
Comparison of Plaque Reduction neutralization to pseudovirus neutralization or ELISA binding. Panel (**A**): Comparison of 50% plaque reduction neutralization titres (PRNT) using LASV lineage II (0043/LV/14) versus PRNT using LASV lineage IV (Josiah) at Biosafety level-4. Panel (**B**): Comparison of 50% reciprocal neutralization titres using LASV pseudoviruses (pv) expressing the glycoprotein complex (GPC) of LASV lineage II versus LASVpv expressing GPC of LASV lineage IV (Josiah). Panel (**C**): Comparison of PRNT reciprocal titres using LASV lineage II to LASV pseudoviruses expressing LASV lineage II GPC reciprocal neutralization titres. Panel (**D**): Comparison of PRNT reciprocal titres using LASV lineage IV to LASV pseudoviruses expressing LASV lineage IV GPC reciprocal neutralization titres. Panel (**E**): Comparison of PRNT reciprocal titres using LASV lineage II to 50% reciprocal binding titres to prefusion glycoprotein (Pf-GP) of LASV lineage II. Panel (**F**): Comparison of PRNT reciprocal titres using LASV lineage IV to 50% reciprocal binding titres to Pf-GP of LASV lineage IV. Panel (**G**): Comparison of LASV pseudoviruses expressing lineage II GPC reciprocal titres to 50% reciprocal binding titres to Pf-GP of LASV lineage II. Panel (**H**): Comparison of LASV pseudoviruses expressing lineage IV GPC reciprocal titres to 50% reciprocal binding titres to Pf-GP of LASV lineage IV. Data was analyzed using Microsoft Excel (version 16.39, Microsoft, Redmond, WA) and JMP software (version 13.0.0, SAS Institute, Inc., Cary, NC). The figure was compiled using Adobe Illustrator (version 15.1.0, San Jose, CA). Note that multiple samples had the same 50% reciprocal titres producing overlapping data points.

A subset of samples (n = 20) was further evaluated in additional neutralization assays and these results were compared to the results of ELISA binding assays (Fig. 8). For this subanalysis plaque reduction neutralization tests (PRNT) at Biosafety Level-4 (BSL-4) were compared to 50% binding titres using pseudoviruses expressing GPC or binding to recombinant Pf-GP. Infectious LASV of lineage II and lineage IV showed a significant correlation (R^2^ = 0.73, P < 0.001) when compared in PRNT (Fig. 8A). A comparison of neutralization titres using LASVpv expressing either the GPC of LASV lineage II or IV on an HIV-1 core also showed a significant correlation (R^2^ = 0.30, P = 0.012). LASVpv neutralization assays were more sensitive than the PRNT, however, there were significant correlations between these neutralization assays. PRNT using LASV of LII showed a significant correlation (R^2^ = 0.40, P = 0.003) to neutralization titres using LASVpv expressing lineage II GPC (Fig. 8C). Similarly, PRNT using LASV lineage IV showed a significant correlation (R^2^ = 0.53, P < 0.001) to neutralization titres using LASV expressing lineage IV GPC. Although less sensitive, LASVpv using a vesicular stomatitis virus core showed significant correlations with neutralization of LASVpv using a HIV core (R^2^ = 0.40, P = 0.001) and with PRNT (R^2^ = 0.37, P = 0.004) (Fig. S4). PRNT titres were also lower than the binding titres to LASV Pf-GP. Significant correlations between PRNT titres and binding titres to LASV Pf-GP of lineage II (Fig. 8E) or lineages IV (Fig. 8F) were not observed. Likewise, significant correlations between pv neutralization titres and binding titres to LASV Pf-GP of lineage II (Fig. 8G) or lineages IV (Fig. 8H) were not observed. These results are expected since the majority of antibodies to LASV GP are non-neutralizing^48^.

## Discussion

### LASV infection induces IgG or IgM that cross-reacts with NP or GP of multiple lineages

As an initial step to evaluate the cross-reactivity potential of Lassa vaccine antigen(s) to cover different lineages, we assessed the ability of the human immune system to mount cross-reactive humoral immune responses during natural infections. Humoral responses to LASV proteins by Nigerian and Sierra Leonean Lassa fever survivors are heterogeneous. Lassa survivors may show IgG and/or IgM reactivity to NP and GP or reactivity to only NP or GP. NP, linked-GP, and Pf-GP antigens representing LASV lineages II- IV were shown to be reactive to LASV-specific antibodies produced by both Nigerian and Sierra Leonean survivors. These results suggest that infection with LASV induces IgG or IgM that is able to effectively cross-react with NP or GP of multiple lineages. Reactivity to Z was variable and there was minimal cross-reactivity between Z of lineages II-IV.

These results differ from a previous study that reported that anti-LASV antibodies preferentially react with the antigens of virus strains present in the local areas^34^. Different techniques were used in this prior study including an indirect immunofluorescence assay (IFA) employing cells infected with different LASV strains and a reverse ELISA that utilized lysates from cells infected with different LASV strains. It is possible that differences in the sensitivities of these assays compared to the recombinant antigen ELISAs account for the more extensive cross-reactivity observed in the current study.

### Heterogeneous induction of LASV cross-neutralizing antibodies by Lassa fever survivors

We also assessed the ability of natural LASV infection to induce cross-neutralizing antibodies. As in previous studies^48^, we observed that not all Lassa fever survivors produce LASV neutralizing antibodies. Neutralizing antibody titres when present are lower than binding titres, which is expected since most antibodies to the LASV GP do not neutralize^48^. Infection with LASV lineage II in Nigerians induces strong neutralizing titres to LASVpv lineage II, but neutralization titres to LASVpv lineages III and IV were lower. In contrast, infection with LASV lineage IV in Sierra Leoneans generally induces cross-reactive neutralizing immune responses to LASVpv of lineage II and III . These results suggest that GP of LASV lineage IV may be more effective than GP of LASV lineage II at presenting epitopes that induce broadly neutralizing antibodies.

### Guidance for procedures to quantify humoral aspects of vaccine-induced immunity to LASV of distinct lineages

Recombinant LASV NP of lineages II–IV produced in *E. coli* appear to be appropriate to assess IgG and IgM responses. NP is not expressed on the surface of the LASV virion or infected cells. Therefore, it is unlikely that anti-NP humoral immune responses are involved in protection from LASV infection. However, anti-NP antibodies can serve as a marker for exposure to infectious LASV either prior to or during Lassa vaccine trials conducted in West Africa. NP could be involved in protective cellular immune response to LASV^53–55^. Most of the LASV vaccines in the CEPI portfolio, with the exception of the measles vectored vaccine do not express LASV NP^14,38^. Recombinant Pan-Lassa linked and Pf-GP IgG/IgM ELISA kits are capable of accurate detection of LASV GP-specific IgG and IgM antibody titres. The Pan-Lassa IgG/IgM ELISA has demonstrated sensitivity to Lassa fever in both Sierra Leone and Nigeria. This study confirms that the Pf-GP construct is a superior reagent for detecting serological responses to LASV GP, which are of prime importance for future vaccine studies.

A LASV pseudovirus platform was more sensitive for quantifying neutralization than PRNT, which is based on replication competent LASV in BSL-4. These findings agree with previous studies that compared several virus neutralization platforms in the evaluation of monoclonal antibodies against LASV^48^. Envelope glycoprotein levels, mechanisms of viral entry, transport, fusion, uncoating, pre-nuclear localization of replicative viral nucleic acids, tropism, and specificity versus passive incorporation of heterologous glycoproteins into particles may all contribute in some degree and affect the sensitivity of each neutralization assay platform. Given the imperfect correlation caution, should be employed when extrapolating the results from pseudovirus platforms to replication competent LASV. While a sensitive pv assay is useful for preclinical vaccine development, comparison of the results to BSL-4 assays would be prudent for vaccine trials.

### Challenges in developing Lassa fever correlates of protection

Correlates of Protection (CoP) for Lassa fever and for Lassa fever vaccines have yet to be defined. These CoP might also be different. Cellular immunity appears to be the primary effective arm of the adaptive immune response against LASV during natural infection^56^. High-titred anti-LASV IgG and virus can be simultaneously present in the blood of human Lassa fever patients^3^. Only a subset of Lassa fever survivors produce LASV neutralizing antibodies, and production of neutralizing antibodies is delayed months into convalescence^48,57^. In contrast to the situation during natural infection, passive serum transfer therapy from survivors protects against disease and death in animal models^58,59^. However, the ability of serum to neutralize LASV of particular strains determined the effectiveness of passive antibody transfer in treating LASV-infected nonhuman primates^59^. Furthermore, neutralizing huMAbs have shown high efficacy in passive immunotherapy of LASV-infected guinea pigs and NHPs^60–62^. In NHP models of lethal Lassa fever infection, treatment with cocktails of selected neutralizing huMAbs completely protected animals even when the first treatment was delayed until eight days post infection, a time when severe disease and dysregulation were clinically evident^61^. Moreover, this huMAb cocktail protected NHPs in late stage Lassa fever, highlighting the relevance of humoral immunity in protecting against lethal Lassa fever^60,61^. Even though neutralizing antibodies are not involved in clearing LASV during natural human infection, they could be important for an induced protective immune response. These studies educate future approaches toward evaluation of the quantity and quality of the humoral response generated by vaccination with LASV specific antigens, namely the viral glycoprotein. The conformation and presentation of the viral glycoprotein is critical to the level of protection^63^. Previous studies demonstrated that multiple doses of inactivated LASV failed to protect NHP from lethal challenge, despite induction of a substantial antibody response^64^. It remains to be determined if protective neutralizing antibodies could be elicited by vaccination, perhaps with engineered forms of LASV GP^63^.

### Induction of cross-protective immune responses by Lassa vaccines

A limited number of LASV vaccine studies have evaluated whether a cross-protective immune response is induced. Safronetz and coworkers^65^ demonstrated that recombinant VSV expressing GP of LASV LIV (Josiah) protects guinea pigs from infection and disease following challenge with LASV isolates originating from Liberia (lineage IV), Mali (lineage V) and Nigeria (lineage I). A recent study in nonhuman primates found that immunization with VSV expressing GPC of LIV (Josiah) as part of a quadrivalent vaccine against Ebola, Sudan, Marburg and Lassa viruses induces a protective immune response against LASV of lineage II^46^. Both cellular immune response and neutralizing antibodies against LASV were induced by the quadrivalent vaccine. Most neutralizing human monoclonal antibodies derived from Sierra Leonean Lassa fever survivors were effective at neutralizing pv expressing LASV GP of divergent lineages (I–IV)^48^. Coupled with the current observation that natural infection with lineage IV viruses (but not lineage II LASV) induces a broad cross-reactive and cross-neutralizing response suggests that it may be possible to induce an immune response with a LASV Josiah (lineage IV) GP-based vaccine that will be cross-protective for infections with LASV of other lineages. Further study on the differences in the induced immune responses to LASV in Nigerians and Sierra Leoneans is warranted and has important implications for vaccine development.

### Limitations to the current study

Representative strains from each of the three most prevalent lineages (II–IV) were selected, but a variety of sublineages (clades) exist within each lineages^20,23^. Due to the high heterogeneity among LASV lineages, continuous monitoring of its mutational spectrum and evolutionary change will be critical for maintaining effective vaccines. The large number of assays employed in this study required the use of a limited number of samples with sufficient volume to comprehensively evaluate binding and neutralizing activities.

### Implications of Lassa diversity for vaccine development

The genetic diversity of LASV presents potentially a major challenge to development of Lassa fever vaccines^14^. A Lassa vaccine or vaccines should provide protective immunity against LASV from the multiple lineages present across West Africa. A variety of Lassa vaccines are under development^14,15,38^. The different vaccine constructs and delivery platforms will present LASV antigens in different ways than natural infection. This study of cross-reactivity and cross-neutralization is based on natural infection to evaluate the cross-protective potentials of Lassa proteins as vaccine candidates. More relevant for vaccine evaluation is of course studies of sera after vaccination and their cross-neutralizing/functional capacity that can be evaluated and compared among different vaccine candidates. Such studies should be done in a similar fashion to the present study, but with sera from vaccinees. Human trials of Lassa fever vaccines based on a protein (or proteins) of a single lineage, such as LASV Josiah (lineage IV), should quantify cross-reactive and cross-neutralizing immune responses to LASV proteins of other lineages and then, of even larger importance, how well the vaccinee sera neutralizes wild-type viruses from different lineages.

## Methods

### Recombinant protein representing LASV lineages II–IV

LASV NPs are produced in *E. coli* using strains engineered for stability and overexpression of genes containing rare codons^39^. Recombinant LASV NPs are initially purified via affinity chromatography, and polished by ion exchange (IEX) chromatography and size exclusion chromatography (SEC), as appropriate. The proteins are then visualized on SDS-PAGE for purity and subjected to Western blot for identity and purity. Recombinant linked and Pf-GP are produced from stable Drosophila S2 cell lines, under serum-free conditions^49^. Resulting proteins are purified via streptactin affinity columns and are > 95% pure following this single step purification. Z proteins are HIS-tagged and purified on Ni–NTA columns.

### IgM and IgG ELISA

The ReLASV Pan-Lassa NP-specific IgM and IgG ELISA utilize microwell plates coated with a mixture of recombinant nucleoprotein NP from lineages II, III, and IV and performed according to the manufacturer’s recommendations^66^. ELISAs utilizing recombinant LASV antigen (NP, linked GP, Pf-GP and Z from LII–IV were performed similarly. LASV antigens (either singular or combined lineages) were coated at 200 to 500 ng/well in 96-well microtiter plates (Nunc A/S, Denmark) using Carb-Bicarb buffer, pH 9.6. After antigens were immobilized, the coated microwell plates were stabilized using proprietary blocking solution, dried and packaged with desiccant.

For the ELISA, lyophilized human monoclonal calibrator and negative control plasma were reconstituted with 0.25 mL laboratory-grade water. Calibrator was diluted 1:101 (0.01 mL/1.0 mL followed by four threefold serial dilutions to create a calibration curve for antibody concentration estimation. For Z protein ELISA screening a well characterized LF convalescent sample with sufficient anti-Z antibody titer was used as assay Reference and similarly diluted to create Reference curve dilutions. LF patient samples were diluted 1:101 in provided Sample Diluent prior to assay. Calibrator (or Reference) dilutions, diluted Negative Control and patient samples were transferred (0.1 mL/well) in duplicate wells. Microwell plates were incubated at ambient temperature (18–30 °C) for 30 min. Microwell plates were washed four times with PBS-Tween wash buffer. Anti-Hu IgG or IgM-horseradish peroxidase conjugate reagent (Jackson ImmunoResearch, West Grove, PA, USA) was added to microwell plate (0.1 mL/well) followed by a 30 min incubation at ambient temperature. After repeating the PBS-Tween wash, 3,3′,5,5′-Tetramethylbenzidine (TMB) Substrate (Moss Biotech Inc., Hanover, Maryland, USA) was added to each well (0.1 mL/well). The TMB substrate was incubated for 10 min followed by addition (0.1 mL/well) of Stopping Solution (0.36 N Sulfuric Acid). Developed ELISA plates were read at 450 nm (with 650 nm reference). IgG or IgM estimated concentration was calculated from Calibrator/Reference Curve plot using 4-paramenter logistic fit. Negative cut-off was determined as the 95^th^ percentile distribution of the study population.

### Pseudovirus assay

LASV pseudoviruses (LASVpv) were generated by co-transfection of HEK293T cells with LASV GPC plasmids and pSG3Denv encoding the envelope-deficient core of HIV-1 as previously described^48^. The pseudoviruses express LASV GPC of different lineages on a particle containing the core proteins of HIV and are capable of a single round of replication. LASV pseudoviruses (LASVpv) capable of a single round of replication were produced by co-transfection of HEK293T cells with LASV GPC plasmids (lineages II–IV) and pSG3Denv, encoding the envelope-deficient core of HIV-1. These LASVpv were assayed in TZMbl cells, a HeLa cell derivative that contains integrated luciferase and β-galactosidase genes under regulatory control of an HIV-1 long terminal repeat, which is activated by HIV-1 Tat after virion entry^67^. The neutralizing activities of Sierra Leonean and Nigerian plasma samples were determined by incubating mixtures of LASVpv stocks at predetermined optimal dilutions with serial dilutions of each plasma sample. Luciferase activity in the cultures was measured at 48 or 72 h after inoculation using a commercially available kit (Promega BriteGlo). Luminescence was measured with a Wallac 1,420 Multilabel Counter (PerkinElmer, Waltham, MA). Studies were also undertaken with LASVpv expressing the core proteins of vesicular stomatitis virus.

### Plaque reduction neutralization test

To assess neutralizing activity of plasma or serum samples, a standard PRNT with infectious LASV was performed under BSL-4 conditions^48,61,62,68^. 100 plaque-forming units of LASV were incubated for 60 min with serial twofold dilutions of each sample in Earle’s Minimal Essential Medium, 1% Pen/Strep and glutamax containing 10% guinea pig complement (Rockland, Limerick, PA). After incubation these reaction mixtures were assayed for residual infectivity (plaque-forming units). The mixture was used to inoculate Vero 76 cells for 60 min. The inoculum was removed and cells were overlaid with agar-containing medium and incubated for 4–6 days at 37 °C. Cells were stained with neutral red for 24 h and plaques were counted. End-point titres were determined through standard 4-parameter logistic regression analysis and represented as the dilution of antibody that neutralized 50% of the plaques. For these assays we used a low-passage LASV Josiah virus stock (lineage IV), which was kindly provided by Tom Ksiazek (University of Texas Medical Branch-Galveston, TX), and originated from CDC Lassa-Josiah CDC number 057562. This strain was originally isolated from a human clinical specimen that was passed once in Vero cells and twice in Vero E6 cells. The study material was the second passage of a lineage II LASV isolate (0043/LV/14) originated from human serum collected from a 30-year-old male patient in Edo State, Nigeria who died in January of 2014^46^.

### Data analysis and statistical methods

Laboratory data, including absorbance values were expressed as mean ± standard error of the mean.Data were analyzed in their individual forms and were not transformed.Two-sample t testswere used to compare absorbance measures between Lassa fever case and control groups.Ordinary linear regression models were used to compare continuous measures between lineage groups and optical density values between ELISA approaches.Pearson’s correlation coefficients or coefficients of determination were used to quantify the magnitude of linear association where linear regression approaches were used.Data was analyzed using Microsoft Excel (version 16.39, Microsoft, Redmond, WA), JMP software (version 13.0.0, SAS Institute, Inc., Cary, NC) and Prism (version 6.07, GraphPad Software, Inc., San Diego, CA). Figures were compiled using Adobe Illustrator (version 15.1.0, San Jose, CA). Analyses were two-tailed with a significance threshold set at p < 0.05.

### Ethics approvals

All methods were carried out in accordance with relevant guidelines and regulations, including the Declaration of Helsinki. All subjects enrolled in this study and/or their legal guardians provided written informed consent. Human subjects testing, incuding the use of excess clinical samples (deidentified, surplus diagnostic samples) under a waiver of consent, was approved by the Tulane University Institutional Review Board (191330), the Nigerian National Health Research Ethics Committee and Irrua Specialist Teaching Hospital (ISTH/HREC/20170915/22) and the Sierra Leone Ethics and Scientific Research Committee (070716). Plasma or serum samples were obtained from Nigeria Lassa fever survivors, contacts, and suspected Lassa fever cases presenting to ISTH, FMC Owo, and FMC Abakaliki between November 2017 and January 2019. In Sierra Leone plasma or serum samples from post-acute, convalescent and Lassa fever survivors were selected from a biorepository of samples collected between October 2012 and January 2014 and between January 2016 and November 2018. Only Nigerian or Sierra Leonean medical personnel staff were involved in the administration of health care to suspected Lassa fever patients. All medical decisions, including whether or not to administer ribavirin to patients, were at the sole discretion of the attending physicians.

## Supplementary information

Supplementary information.
