# Supplementary material for: Antibodies from Sierra Leonean and Nigerian Lassa fever survivors cross-react with recombinant proteins representing Lassa viruses of divergent lineages
Source: Sci Rep. 2020 Sep 29;10:16030. doi: 10.1038/s41598-020-72539-w (PMC7525497; doi:10.1038/s41598-020-72539-w)
Supplement: Supplementary file 1 — Supplementary information. [file 41598_2020_72539_MOESM1_ESM.docx]

**SUPPLEMENTAL MATERIAL**

**Antibodies from Sierra Leonean and Nigerian Lassa fever survivors cross-react with recombinant proteins representing Lassa viruses of divergent lineages**

Megan L. Heinrich^1^, Matthew L. Boisen^1^, Diana K. S. Nelson^1^, Duane J. Bush^1^, Robert W. Cross^2,3^, Anatoliy P. Koval^1^, Andrew R. Hoffmann^4^, Brandon J. Beddingfield^4^, Kathryn M. Hastie^5^, Megan M. Rowland^1^, Irina Aimukanova^1^, Sophia Koval^1^, Raju Lathigra^1^, Viktoriya Borisevich^3,6^, Mambu Momoh^7,8,9^, John Demby Sandi^8^, Augustine Goba^8^, lkponmwosa Odia^10^, Francis Baimba^8^, John O. Aiyepada^10^, Benevolence Ebo^10^, Philomena Eromon^11,12^, Chinedu Ugwu^11,12^, Onikepe Folarin^11,12^, Testimony Olumade^11,12^, MacDonald N. Onyechi^13^, Johnson Etafo^13^, Rashidat Adeyemi^14^, Elijah E. Ella^14^, Maryam Aminu^14^, Simji S. Gomerep^15^, Matthew Afam Eke^16^, Olusola Ogunsanya^17^, George O. Akpede^10,18,19^, Danny O. Asogun^10,20^, Sylvanus A. Okogbenin^10,21^, Peter O. Okokhere^10,22,23^**,**  Johan Holst^24^, Jeffrey G. Shaffer^25^, John S. Schieffelin^26^, Thomas W. Geisbert^2,3^, Erica Ollmann Saphire^5^, Christian T. Happi^11,12,^ *, Donald S. Grant^8,9,^*, Robert F. Garry^1,4,^* and Luis M. Branco^1^

^1^ Zalgen Labs, LCC, Germantown, MD, USA

^2^Department of Microbiology and Immunology University of Texas Medical Branch at Galveston, Galveston, TX, USA

^3^Galveston National Laboratory, Galveston, TX, USA

^4^ Tulane University, School of Medicine, Department of Microbiology and Immunology, New Orleans, LA, USA

^5^La Jolla Institute for Immunology, La Jolla, CA 92037, USA

^6^Department of Pathology, University of Texas Medical Branch at Galveston, Galveston, TX, USA

^7^Eastern Polytechnic Institute, Kenema, Sierra Leone

^8^Viral Hemorrhagic Fever Program, Kenema Government Hospital, Kenema, Sierra Leone

^9^Ministry of Health and Sanitation, Freetown, Sierra Leone

^10^Institute of Lassa Fever Research and Control, Irrua Specialist Teaching Hospital, Irrua, Edo State, Nigeria

^11^The African Center of Excellence for Genomics of Infectious Diseases, Redeemer’s University, Ede, Osun State, Nigeria

^12^Department of Biological Sciences, College of Natural Sciences, Redeemer’s University, Ede, Osun State, Nigeria

^13^Federal Medical Center Owo, Owo, Nigeria

^14^Ahmadu Bello University, Zaria, Nigeria

^15^University Teaching Hospital, Jos, Nigeria

^16^Federal Medical Center, Abakaliki, Abakaliki, Nigeria

^17^University of Ibadan, Ibadan, Nigeria

^18^ Department of Paediatrics, Irrua Specialist Teaching Hospital, Irrua, Nigeria.

^19^ Department of Paediatrics, College of Medicine, Ambrose Alli University, Ekpoma, Nigeria

^20^Department of Community Medicine, Irrua Specialist Teaching Hospital, Irrua, Nigeria.

^21^Department of Obstetrics and Gynaecology, Irrua Specialist Teaching Hospital, Irrua, Nigeria.

^22^The Department of Medicine, Irrua Specialist Teaching Hospital, Irrua, Edo State, Nigeria

^23^The Department of Medicine, Faculty of Clinical Sciences, Ambrose Alli University, Ekpoma, Edo State, Nigeria

^24^CEPI (Coalition for Epidemic Preparedness Innovations), Oslo, NORWAY*.*

^25^ Department of Biostatistics and Bioinformatics, Tulane School of Public Health and Tropical Medicine, New Orleans, Louisiana, United States of America, USA

^26^Sections of Infectious Disease, Departments of Pediatrics and Internal Medicine, School of Medicine, Tulane University, New Orleans, LA, USA

***Corresponding Authors**

Christian T. Happi, PhD

Department of Molecular Biology and Genomics

Center of Excellence for Genomics of Infectious Diseases (ACEGID)

Directorate of Research Innovations and Partnerships (DRIPs)

Redeemer's University

Ede, Osun State, Nigeria

[happic@run.edu.ng](mailto:happic@run.edu.ng)

Donald S. Grant, M.B.Ch.B. MPH

Viral Hemorrhagic Fever Program, Kenema Government Hospital, Kenema

Ministry of Health and Sanitation, Freetown

Sierra Leone

donkumfel@yahoo.co.uk

Robert F. Garry, PhD

Department of Microbiology and Immunology

Tulane University School of Medicine

1430 Tulane Avenue, JBJ568

New Orleans, LA 70118

504-988-2027

[rfgarry@tulane.edu](mailto:rfgarry@tulane.edu)

**
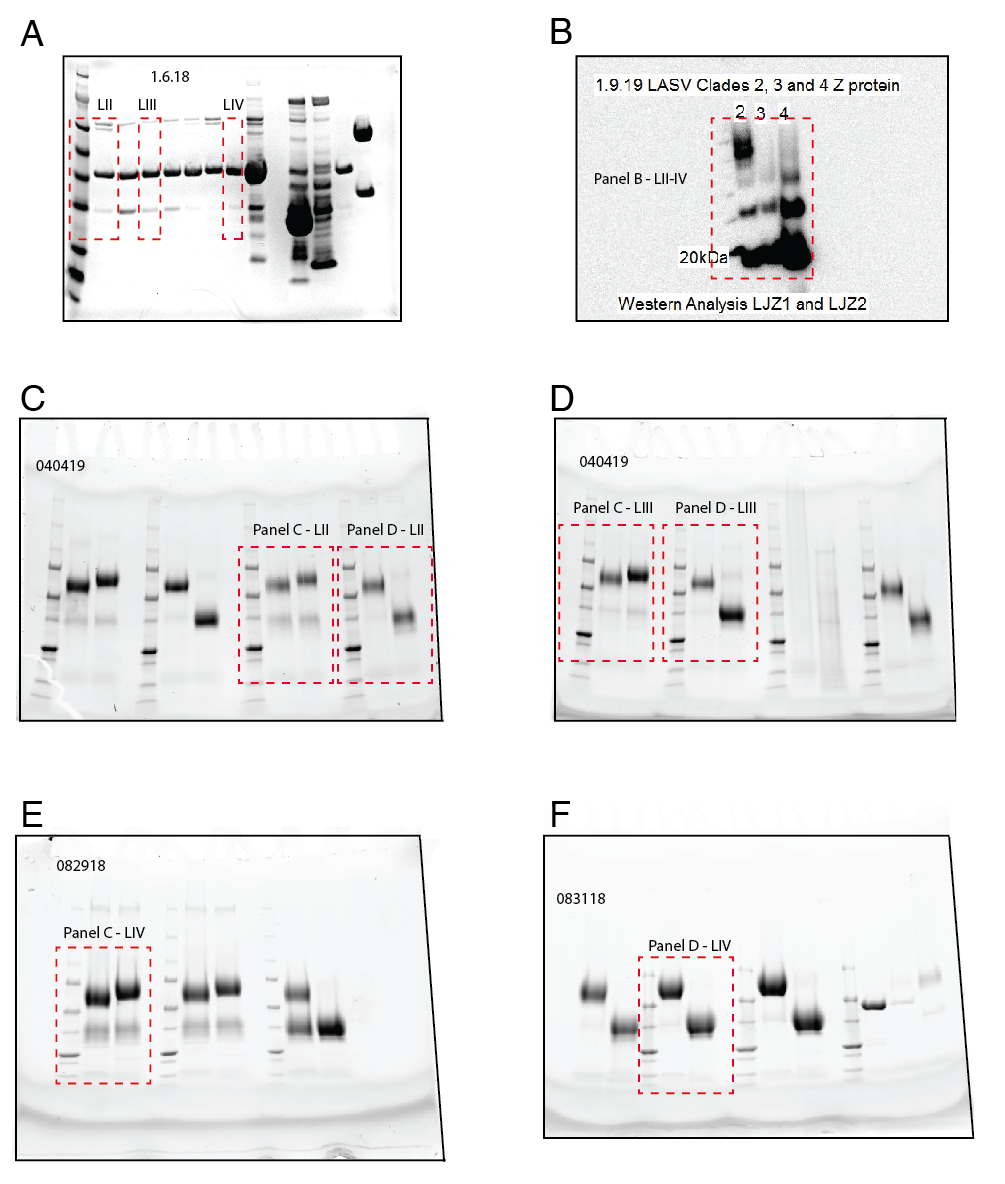
**

**Supplemental Figure 1. Uncropped polyacrylamide gels and western blot.**  Original unfiltered images of gels and a western blot used to assamble Fig. 1 (dates are indicated). Red dashed boxes indicate lanes cropped to assemble indicated panels. Panel A: Simply Blue stained gel showing representative lots and purification intermediates for LASV nucleoprotein of lineages II, III and IV (LII-IV) shown in Fig. 1 A. Panel B: western blot showing representative LASV Zinc protein lots (LII-IV). shown in Fig. 1 B. Panels C-F: Stain Free gels of linked glycoprotein (Fig. 1C) or prefusion glycoprotein (Fig. 1D) from LII-LIV as indicated. The figure was compiled using Adobe Illustrator (version 15.1.0, San Jose, CA).

**
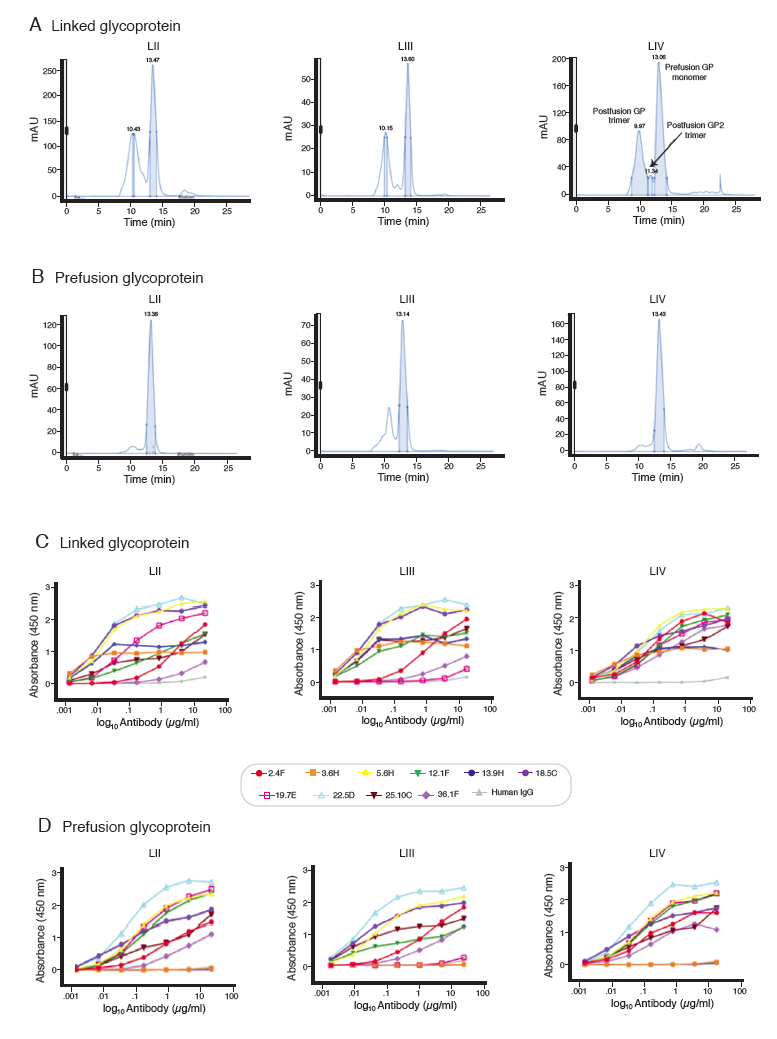
**

**Supplemental Figure 2. Analyses of Lassa virus glycoproteins from lineages II-IV.**  Lassa viruses linked glycoprotein (Panel A) or prefusion glycoprotein (Panel B) from lineages II-IV (LII, LIII, LIV) were analyzed by size exclusion chromatography-multiangle light scattering. Binding of a panel of human monoclonal antibodies to linked glycoprotein (Panel C) or prefusion glycoprotein (Panel D) was quantified by ELISA. The figure was compiled using Adobe Illustrator (version 15.1.0, San Jose, CA).


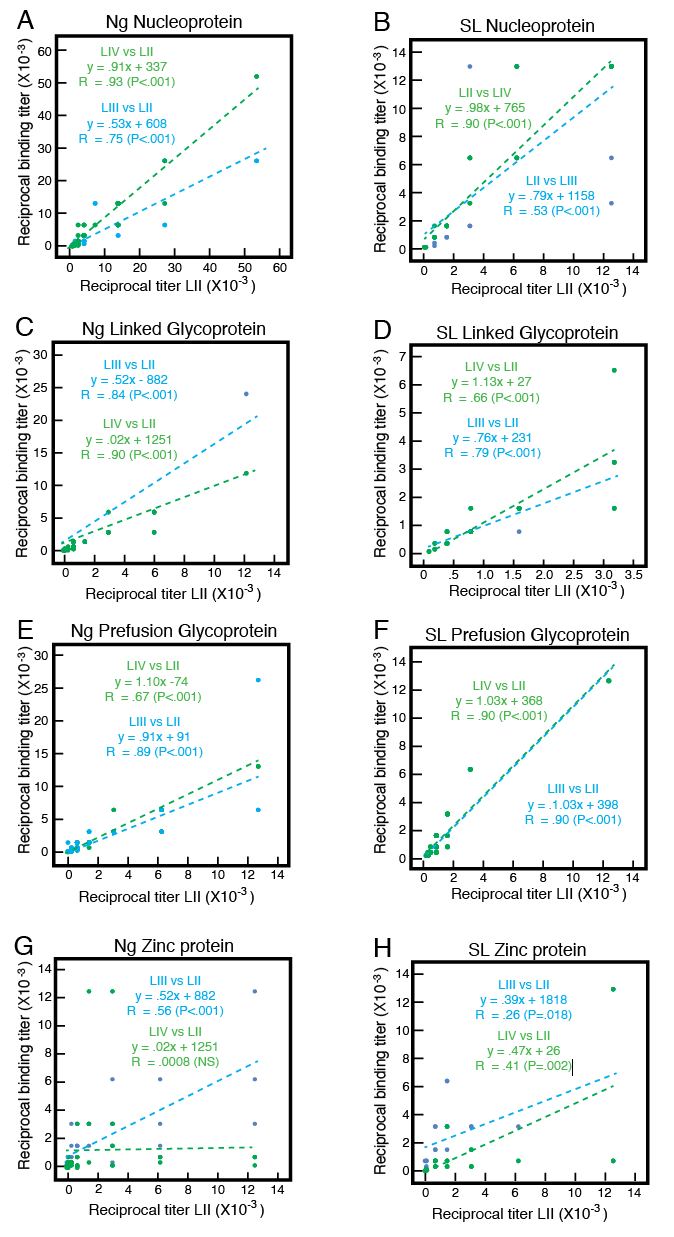


**Supplemental Figure 3. Cross-reactivity of IgG in plasma and serum from Lassa fever survivors for recombinant proteins from LASV of lineages II-IV determined using endpoint titres.**  Endpoint titres for binding of IgG in serum or plasma samples (1:100 dilution) from Nigerian (NG, Panels A, C, E, G; n = 40) or Sierra Leonean (SL, Panels B, D, F, H; n = 61) Lassa fever survivors to NP or Pf-GP from LASV representing lineages III-IV were quantified using ELISA individually coated individually with NP, linked GP, Pf-GP or Z from LASV lineages II-IV as indicated. Green dotted lines are linear regression plots of seroreactivity of lineage II (LII) LASV antigens versus lineage III (LIII) antigens. Data was analyzed using Microsoft Excel (version 16.39, Microsoft, Redmond, WA) and JMP software (version 13.0.0, SAS Institute, Inc., Cary, NC). The figure was compiled using Adobe Illustrator (version 15.1.0, San Jose, CA). Blue dotted lines are linear regression plots of seroreactivity of LII LASV antigens versus lineage IV (LIV) antigens.

**
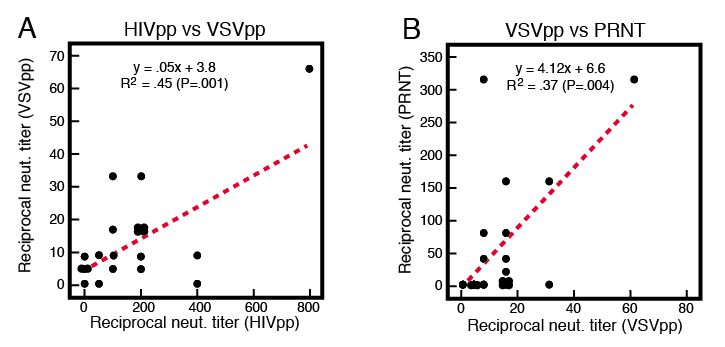
**

**Supplemental Figure 4. Comparison of neutralization with LASV pseudovirus with two different cores and comparison of a LASV pseudovirus with a vesicular stomatitis virus core to the LASV plaque reduction neutralization test.** Panel A: Comparison of 50% reciprocal neutralization titres using LASV pseudovirus with a human immunodeficiency virus core (HIVpv) versus LASV pseudovirus with a vesicular stomatitis virus core (VSVpv). Both pseudoviruses expressed the glycoprotein complex (GPC) of LASV lineage IV (Josiah). Panel B: Comparison of 50% reciprocal neutralization titres using LASVpv with a VSV core expressing GPC of LASV lineage IV versus plaque neutralization reduction test (PRNT) reciprocal titres using LASV lineage IV (Josiah). Data was analyzed using Microsoft Excel (version 16.39, Microsoft, Redmond, WA) and JMP software (version 13.0.0, SAS Institute, Inc., Cary, NC). The figure was compiled using Adobe Illustrator (version 15.1.0, San Jose, CA). Note that multiple samples had the same 50% reciprocol titres producing overlapping data points.
